# Machine Learning-Based Prediction Method for Tremors Induced by Tacrolimus in the Treatment of Nephrotic Syndrome

**DOI:** 10.3389/fphar.2022.708610

**Published:** 2022-04-27

**Authors:** Bing Shao, Youyang Qu, Wei Zhang, Haihe Zhan, Zerong Li, Xingyu Han, Mengchao Ma, Zhimin Du

**Affiliations:** ^1^ Department of Pharmacy, The Second Affiliated Hospital, Harbin Medical University (Key Laboratory of Medications Research, College of Heilongjiang Province), Harbin, China; ^2^ School of Pharmacy, Harbin Medical University, Harbin, China; ^3^ Neurology Department, The Second Affiliated Hospital, Harbin Medical University, Harbin, China; ^4^ Nephrology Department, The Second Affiliated Hospital, Harbin Medical University, Harbin, China; ^5^ State Key Laboratory of Quality Research in Chinese Medicines, Macau University of Science and Technology, Macau, China

**Keywords:** tremor, tacrolimus, nephrotic syndrome, machine learning model, recursive feature elimination, neural network

## Abstract

Tremors have been reported even with a low dose of tacrolimus in patients with nephrotic syndrome and are responsible for hampering the day-to-day work of young active patients with nephrotic syndrome. This study proposes a neural network model based on seven variables to predict the development of tremors following tacrolimus. The sensitivity and specificity of this algorithm are high. A total of 252 patients were included in this study, out of which 39 (15.5%) experienced tremors, 181 patients (including 32 patients who experienced tremors) were randomly assigned to a training dataset, and the remaining were assigned to an external validation set. We used a recursive feature elimination algorithm to train the training dataset, in turn, through 10-fold cross-validation. The classification performance of the classifer was then used as the evaluation criterion for these subsets to find the subset of optimal features. A neural network was used as a classification algorithm to accurately predict tremors using the subset of optimal features. This model was subsequently tested in the validation dataset. The subset of optimal features contained seven variables (creatinine, D-dimer, total protein, calcium ion, platelet distribution width, serum kalium, and fibrinogen), and the highest accuracy obtained was 0.8288. The neural network model based on these seven variables obtained an area under the curve (AUC) value of 0.9726, an accuracy of 0.9345, a sensitivity of 0.9712, and a specificity of 0.7586 in the training set. Meanwhile, the external validation achieved an accuracy of 0.8214, a sensitivity of 0.8378, and a specificity of 0.7000 in the validation dataset. This model was capable of predicting tremors caused by tacrolimus with an excellent degree of accuracy, which can be beneficial in the treatment of nephrotic syndrome patients.

## Introduction

Tacrolimus is a profoundly effective immunosuppressive drug that blocks calcineurin-mediated T-cell activation by binding to the immunophilin FKBP12 (Yamauchi et al., 2004). It is used to prevent allograft rejection in solid organ transplantation. In recent years, it has also been widely used for further immunosuppression in immune-mediated glomerular diseases, particularly in steroid-dependent and steroid-refractory nephrotic syndromes (NSs) (Manabe et al., 2018). Clinical applications show that tacrolimus has significant inter- and intra-individual variability in pharmacokinetics and clinical responses. However, tacrolimus treatment is associated with various adverse reactions (ADRs), including acute and chronic nephrotoxicities, neurotoxicities, hypertension, post-transplant diabetes mellitus, gastrointestinal manifestations, and hyperlipidemia (Campagne et al., 2019). Several studies have reported that tacrolimus-related ADRs were more frequent or severe at higher tacrolimus exposures. The frequency and severity of neurotoxicities and several other ADRs have been reduced by adhering to dose minimization protocols for tacrolimus drug exposure after a transplant (Ali, 2013; Campagne et al., 2019).

However, a tremor as a mild neurotoxic manifestation was the most common ADR, affecting 4.5–56% of patients (from SIDER 4.1: Side Effect Resource-http://sideeffects.embl.de/). A reduction in the dosage of tacrolimus is not sufficient to reduce or prevent tremors. Patients have often complained of tremors even during treatment of NS with low doses of tacrolimus (0.05–0.1 mg/kg/d). There are no data on the usage of tacrolimus in the treatment of NS and the consequent occurrence of tremors. The tremors affected both upper and lower limbs and were considered to be induced by movement; however, in approximately 50% of the cases, tremors occurred during movement and rest (Erro et al., 2018). Such tremors significantly affect the work and personal lives of NS patients, and patients would not be able to tolerate tacrolimus treatment.

In recent years, a machine learning model was used to predict the occurrence of ADRs after a drug was administered. This model could obtain the characteristic variables related to the occurrence of ADRs and identify high-risk patients prone to some type of ADRs early on to change treatment strategies (Tangiisuran et al., 2014; van Dijkhuizen et al., 2015; Yin et al., 2017). Studies on tacrolimus-induced tremors are limited, and there are many unanswered questions, especially in cases where tacrolimus was administered in small doses. One study had suggested that the cholesterol plasmatic concentration may play a role in predicting the occurrence of tremors in kidney-transplant patients on tacrolimus therapy, but further research is necessary for verification (Erro et al., 2018). This study presents a good model to assess the predictive factors for tremors induced by tacrolimus therapy for NS patients. At the same time, this study is also the first to use seven variables. The tremors that patients undergoing tacrolimus therapy commonly experience are debilitating, and their prediction can aid in providing better medical treatment to nephrotic syndrome patients.

## Materials and Methods

### Patients and Study Design

From January 2013 to December 2017, a total of 341 patients were diagnosed with NS, and they were initially treated with tacrolimus (0.05 mg/kg, the daily dose was not greater than 4 mg, and the blood concentration was not greater than 8 ng/ml). All patients received correct medication instructions and follow-up from clinical pharmacists. The ADRs experienced by the patients, including tremors, were recorded during these follow-ups. Eventually, 252 patients could take tacrolimus for more than 3 months and had more than one follow-up within 6 months. We subsequently performed a retrospective single-center study using the data from the aforementioned patients, which were available on the electronic medical record system at the Second Affiliated Hospital of Harbin Medical University. The detailed demographics and baseline clinical characteristics were collected for further study before the patients were treated with tacrolimus.

### Ethics Approval and Consent to Participate

The protocols used in this study were approved by the Medical Ethical Committee of the Second Affiliated Hospital of Harbin Medical University (No. KY 2017-242). All subjects were anonymized, and the ethics committee approved the waiving of informed consent. This study has conformed to the ethical guidelines of the 1975 Declaration of Helsinki.

### Definition

In this study, NS is defined as a group of clinical syndromes, including macroalbuminuria (adult>3.5 g/d), hypoproteinemia (<30 g/L), obvious edema, and/or hyperlipidemia (Nishi et al., 2016).

### Statistical Analysis

Continuous variables were presented as mean values with a 95% confidence interval, and the categorical variables were expressed as the number and corresponding percentage of each category (e.g., male). A *t*-test was used to compare if the mean values of two groups were significantly different. Meanwhile, a z-test was utilized to compare the proportion differences within categorical variables. A 2-tailed value of *p* < 0.05 was established as the threshold of statistical significance. All data analyses were performed using R language (ver 3.6.3).

### Data Preprocessing

In this study, records that were missing more than 50% of the feature values were considered noise data and deleted from the dataset. We also deleted records where more than 20% of the feature values were missing because there were too much missing data to all them. For the remaining missing values, we used multivariate imputation via the chained equations method.

The feature indices of the patients were of different dimensions or dimension units. This caused data with different attributes to be in different orders of magnitude. This might have, in turn, caused a few indicators to be ignored, which affected the results of the prediction model. After normalization using the common min–max standardization, all attributes of the original data were normalized to a (0, 1) range.

### Prediction Model Development

In this study, a prediction model based on machine learning was developed. The recursive feature elimination (RFE) method was used for feature selection, which was combined with a neural network (NN) to form the classifiers to build the prediction model.

The selected patients were randomly divided into two separate data sets: 70% of the patients from our database were assigned to the training dataset (the algorithm creation group) and the remaining 30% were reserved as the external validation set (validation group) to obtain unbiased estimates of accurate classification rates and variable importance.

RFE is a greedy algorithm used to find the optimal subset of features. It searched the complete set as a starting point, and the prediction accuracy after 10-fold cross-validation was used for the estimation principle. After ranking, the most related features ranked the highest, and the lowest ranking features were assigned negative values at the end of the iteration. In this study, the steps of the algorithm were as follows: ① initialize the feature set *H*; ② select the classifier NN; ③ calculate the weight of each feature, *h*_i_, in *H* (the principle gives the accuracy of NN); ④ delete the minimum weight feature, *h*_j_, and update *H*; ⑤ repeat steps 3 and 4 until *H* has only one feature left; and ⑥ perform feature importance ranking (Chang et al., 2019).

The RFE-NN method can export a list in the decreasing order of importance of the features. From this sorted list, we could obtain a set of feature subsets H1 ⊂ H2 ⊂ Hn (*n* indicates the number of features included in the list; in this study, *n* is 64). *H*_1_ was composed of the first feature, *H*_2_ consisted of the top two features in the list, and so on. *Hn* represented the complete feature set. For a large number of features, RFE with cross-validation (RFECV) could be used to reduce the computation. In the exhaustive method, the number of all the subsets was 2^64^–1; however, the number of subsets that RFECV needed to verify was only 64. The 10-fold cross-validation used in this study was suitable for datasets with fewer data. The samples in the dataset were randomly divided into 10 subsets of mutexes and similar size. During training, nine subsets were chosen, in turn, to form the training set, and the remaining one subset formed the test set. This method allowed the model to be trained and tested 10 times using different training and testing sets. Every test yielded an accurate rate, and we took the average of 10 test results as the final result to evaluate the accuracy of the algorithm. Essentially, RFECV could cross-validate different combinations of features. The sum of the decision coefficients was calculated, and the optimal feature combination was selected based on the importance of different features to the accurate rate.

The single hidden layer NN model that was used to predict tremors consisted of input, hidden, and output layers. The input layer consisted of all the values from the input, which was, in our study, the numerical representation of the seven features selected by RFE. In the hidden layer, every perceptron unit took input from the input layer, subsequently multiplied, and added it to the initially random values. This initial output was activated by the ReLU activation function. The third layer was the output layer, and it took all the perceptrons in the previous layer as inputs, multiplied, and added their outputs to the initially random values. It was then activated by a sigmoid function. This layer outputted a value between 0 and 1, which, in this test, represented the likelihood of a patient developing tremors. The 10-fold cross-validation was also used to tune the prediction model using the number of neurons in the hidden layer as the tuning parameter. The prediction performance was assessed by the corresponding area under the curve (AUC) of the receiver operating curve (ROC) for this model. The equations for accuracy (ACC), sensitivity (SE), and specificity (SP) are as follows:

$$\mathrm{SE=}\frac{\mathrm{TP}}{\mathrm{TP+FN}},$$

$$\mathrm{SP=}\frac{\mathrm{TN}}{\mathrm{TN+FP}},$$

$$\mathrm{ACC=}\frac{\mathrm{TP+TN}}{\mathrm{TP+FP+TN+FN}}.$$

## Results

### Patient Characteristics

A total of 252 patients (98 men and 154 women; mean age of approximately 45 years) were included in this study, of which 39 patients (15.5%) developed tremors and 64 variables, including demographic information and laboratory values, were collected for each patient; 181 patients (of which 32 developed tremors) in our database were assigned to the training dataset; and the remaining 71 patients (of which seven developed tremors) were reserved as the external validation set. The clinical characteristics before normalization of the patients who developed tremors and those who did not in the training dataset before tacrolimus administration are shown in Table 1. There was no significant difference between the baseline values of most variables of the patients who developed tremors and those who did not, except for uric acid (UA) and total protein (TP) (*p* < 0.05).

**TABLE 1:** Demographic and clinical data before normalization in tremor (Train_1) and non-tremor (Train_0) patients of the training data set.

Variable	Train_1_mean	Train_1_conf	Train_0_mean	Train_0_conf	*p* value
Age (years)	42.531	3.999	44.617	2.327	0.441
Weight	66.953	3.554	70.589	2.107	0.138
WBC	7.603	1.047	8.083	0.925	0.645
NEUT_pct	60.094	3.883	61.650	1.891	0.489
LYMPH_pct	31.919	3.662	29.323	1.597	0.181
MONOR_pct	5.375	0.643	5.683	0.343	0.445
EO_pct	2.306	0.762	2.648	0.475	0.534
BASO_pct	0.306	0.148	0.264	0.071	0.618
NEUT	6.284	3.342	4.930	0.467	0.138
LY	2.284	0.324	2.113	0.136	0.303
MONO	0.401	0.074	0.423	0.036	0.610
EO	0.167	0.064	0.184	0.036	0.670
BASO	0.023	0.014	0.876	1.697	0.647
HGB	132.805	10.397	139.642	4.037	0.173
RBC	4.579	0.177	4.806	0.477	0.666
HCT	40.981	1.754	41.586	1.131	0.642
MCV	89.569	2.086	90.274	1.174	0.607
MCH	30.231	0.907	32.791	3.897	0.550
MCHC	337.094	3.689	337.384	4.730	0.956
RDW_CV	13.028	0.330	13.357	0.457	0.516
RDW_SD	42.431	1.017	43.211	0.937	0.459
PLT	256.594	23.475	242.530	11.809	0.315
MPV	10.591	0.398	10.879	0.337	0.448
PDW	13.319	0.814	12.599	0.319	0.069
PCT	0.271	0.026	0.253	0.012	0.210
P_LCR	31.331	2.580	30.109	1.167	0.385
ALT	23.031	4.786	19.792	1.808	0.152
AST	21.875	2.278	22.785	1.901	0.671
AST/ALT	1.259	0.299	1.359	0.136	0.541
γ_GGT	35.156	19.855	46.940	14.510	0.476
ALP	63.906	6.639	74.060	9.920	0.355
TP	47.822	2.461	51.515	1.592	0.045*
ALB	23.534	2.190	25.681	1.397	0.183
GLO	24.266	1.694	25.646	1.015	0.241
ALB/GLO	1.009	0.122	1.063	0.073	0.523
TBIL	7.075	1.567	8.610	1.057	0.206
DBIL	2.381	0.383	2.836	0.386	0.293
IDBIL	4.675	1.241	5.774	0.714	0.187
CHE	11879.250	1238.819	11646.826	588.500	0.741
UREA	5.533	0.665	7.441	1.439	0.229
CREA	63.672	6.436	103.666	20.774	0.081
UREA/CREA	88.474	12.527	77.056	4.982	0.064
UA	331.350	35.333	386.987	17.896	0.009**
Bicarbonate	28.138	1.204	28.919	3.230	0.826
GLU	5.576	0.514	5.343	0.136	0.217
Ka	4.105	0.109	4.813	1.350	0.633
Na	140.834	0.945	138.980	2.629	0.521
Cl	105.425	1.298	105.443	1.637	0.992
Ca	2.033	0.055	2.893	1.368	0.566
P	1.261	0.063	1.347	0.180	0.664
Mg	0.843	0.033	0.861	0.024	0.516
AG	11.184	1.172	11.382	0.703	0.808
UPRO	2.844	0.261	2.906	0.131	0.688
U_RBCH	25.025	12.607	66.632	56.407	0.502
U_WBCH	3.331	1.251	77.895	100.002	0.497
PT	9.725	0.266	9.990	0.238	0.322
PTA	114.813	6.191	112.570	2.630	0.484
PTR	0.908	0.028	0.925	0.022	0.491
INR	0.914	0.026	1.145	0.434	0.628
APTT	35.063	1.711	41.190	8.109	0.491
FIB	4.171	0.294	4.150	0.192	0.925
TT	14.359	0.435	14.083	0.274	0.382
D_Dimer	428.094	301.594	478.054	132.936	0.756
Sex	16	32	53	149	0.185

Note: *p<0.05, **p<0.01. Abbreviations in the table: WBC: white blood cells, NEUT: neutrophile granulocyte, LY: lymphocyte, MONOR: proportion of monocytes, EO: eosinophils, BASO: basophil, _pct: percentage, HGB: hemoglobin, RBC: red blood cells, HCT: hematocrit, MCV: mean corpuscular volume, MCH: mean corpuscular hemoglobin, MCHC: mean corpuscular hemoglobin concentration, RDW_CV: red blood cell volume distribution width, RDW_SD: standard deviation of red blood cell distribution width, PLT: platelet technology, MPV: mean platelet volume, PDW: platelet distribution width, PCT: thrombocytocrit, P_LCR: platelet-large cell rate, ALT: alanine transaminase, AST: aspartic transaminase, γ_GGT: gamma-glutamyltransferase, ALP: alkaline phosphatase, TP: total protein, ALB: albumin, GLO: globulin, TBIL: total bilirubin, DBIL: direct bilirubin, IDBIL: indirect bilirubin, CHE: cholinesterase, UA: uric acid, GLU: glucose, AG: anion gap, UPRO: urine protein, U_RBCH: urinary red blood cell (high magnification), U_WBCH: urinary white blood cell (high magnification), PT: prothrombin time, PTA: prothrombin activity, PTR: prothrombin time ratio, INR: international normalized ratio, APTT: activated partial thromboplastin time, FIB: fibrinogen, and TT: thrombin time.

### Variables of Importance

**FIGURE 1:**
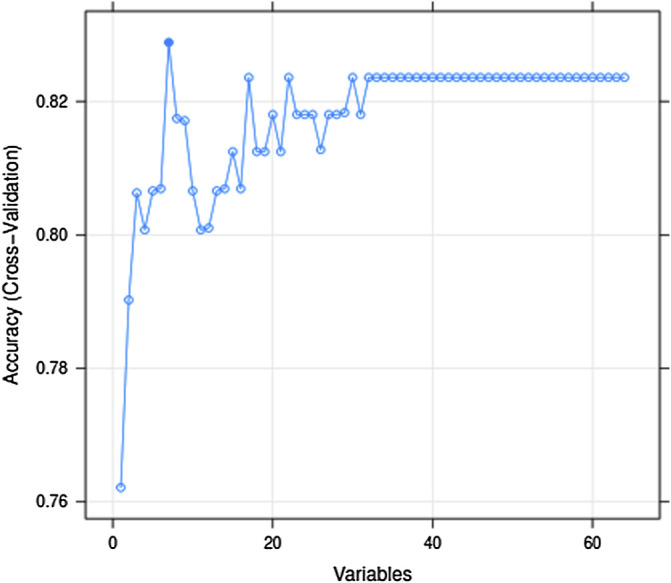
Relationship between the accuracy and the number of variables after cross-validation.

Generally, the error of a model decreases with an increase in the number of variables. However, increasing the number of variables is not suitable for clinical practice. To identify the prominent variables, we carried out feature selection using RFE. There were 64 variables in this study, and all combinations from 1 to 64 should be analyzed to select the subset of optimal features (highest accuracy). The outer resampling method: cross-validated (10-fold) could greatly reduce the computation. The results of the recursive feature selection on the training dataset are shown in Table 2. The accuracy values and kappa coefficients with standard deviations for each subset are also given in Table 2. A more intuitive diagram (Figure 1) shows the relationship between the accuracy and the number of variables after cross-validation. When the number of variables was increased to seven, the highest accuracy was obtained (0.8288). When the number of variables gradually increased from 34 to 64, the accuracy remained constant. Therefore, the final model included seven indispensable features for tremor prediction: creatinine (CREA), D-dimer (D_Dimer), total protein (TP), calcium ion (Ca), platelet distribution width (PDW), serum kalium (Ka), and fibrinogen (FIB). The top five variables (out of seven) were CREA, D_Dimer, TP, Ca, and PDW. Moreover, the changes in the AUC values after excluding each variable were compared to re-evaluate the importance. The larger the change in the AUC value when a certain variable was excluded from the model, the more important the variable was assigned. The classification results were consistent, and there were only a few differences in the sorting of the importance of the variables. The changes in the AUC values when each of the seven variables was excluded from the model are shown in Table 3. Using this method based on the changes in the AUC values, the top five variables (out of seven) are FIB, CREA, Ca, PDW, and Ka. The former approach (Table 2; Figure 1) emphasized more on the importance of all of the variables as a whole to the model; however, the method based on the changes in the AUC values (Table 3) emphasized the impact of individual variables on the model. However, the aforementioned differences did not affect the classification results.

**TABLE 2:** Results of the recursive feature selection performed on the training data set.

Variable	Accuracy	Accuracy SD	Kappa	Kappa SD	Selected
1	0.7621	0.041887	0.08312	0.24635	
2	0.7902	0.095455	0.06158	0.21474	
3	0.8063	0.099982	0.06627	0.24686	
4	0.8007	0.079142	0.04805	0.21157	
5	0.8066	0.084922	0.0463	0.19316	
6	0.8069	0.037175	0.05176	0.20010	
**7**	**0.8288**	**0.137217**	**0.04763**	**0.28717**	*
8	0.8174	0.036749	0.04558	0.20904	
9	0.8171	0.093225	0.03936	0.22117	
10	0.8066	−0.025062	0.03909	0.05452	
11	0.8007	−0.008761	0.04028	0.13650	
12	0.8010	−0.008866	0.04606	0.13657	
13	0.8066	−0.025062	0.03909	0.05452	
14	0.8069	−0.024476	0.03711	0.05369	
15	0.8125	−0.015385	0.03642	0.04865	
16	0.8069	−0.024476	0.03711	0.05369	
17	0.8236	0.030769	0.01823	0.09730	
18	0.8125	−0.015385	0.03642	0.04865	
19	0.8125	−0.015385	0.03642	0.04865	
20	0.8180	−0.009091	0.02282	0.02875	
21	0.8125	−0.015385	0.03642	0.04865	
22	0.8236	0.000000	0.01823	0.00000	
23	0.8180	−0.009091	0.02282	0.02875	
24	0.8180	−0.009091	0.02282	0.02875	
25	0.8180	−0.009091	0.02282	0.02875	
26	0.8128	−0.018286	0.03364	0.03855	
27	0.8180	−0.009091	0.02282	0.02875	
28	0.8180	−0.009091	0.02282	0.02875	
29	0.8183	−.009195	0.03176	0.02908	
30	0.8236	0.000000	0.01823	0.00000	
31	0.8180	−0.009091	0.02282	0.02875	
32	0.8236	0.000000	0.01823	0.00000	
33	0.8236	0.036364	0.03191	0.14969	
34	0.8236	0.000000	0.01823	0.00000	
35	0.8236	0.000000	0.01823	0.00000	
36	0.8236	0.000000	0.01823	0.00000	
37	0.8236	0.000000	0.01823	0.00000	
38	0.8236	0.000000	0.01823	0.00000	
39	0.8236	0.000000	0.01823	0.00000	
40	0.8236	0.000000	0.01823	0.00000	
41	0.8236	0.000000	0.01823	0.00000	
42	0.8236	0.000000	0.01823	0.00000	
43	0.8236	0.000000	0.01823	0.00000	
44	0.8236	0.000000	0.01823	0.00000	
45	0.8236	0.000000	0.01823	0.00000	
46	0.8236	0.000000	0.01823	0.00000	
47	0.8236	0.000000	0.01823	0.00000	
48	0.8236	0.000000	0.01823	0.00000	
49	0.8236	0.000000	0.01823	0.00000	
50	0.8236	0.000000	0.01823	0.00000	
51	0.8236	0.000000	0.01823	0.00000	
52	0.8236	0.000000	0.01823	0.00000	
53	0.8236	0.000000	0.01823	0.00000	
54	0.8236	0.000000	0.01823	0.00000	
55	0.8236	0.000000	0.01823	0.00000	
56	0.8236	0.000000	0.01823	0.00000	
57	0.8236	0.000000	0.01823	0.00000	
58	0.8236	0.000000	0.01823	0.00000	
59	0.8236	0.000000	0.01823	0.00000	
60	0.8236	0.000000	0.01823	0.00000	
61	0.8236	0.000000	0.01823	0.00000	
62	0.8236	0.000000	0.01823	0.00000	
63	0.8236	0.000000	0.01823	0.00000	
64	0.8236	0.000000	0.01823	0.00000	

Note: The meaning of the bold values: when the number of variables was increased to seven, the accuracy was the highest.

**TABLE 3:** Change in the AUC values when each variable is excluded from the model.

Variable	Value of AUC after excluding a variable	Change in the value of AUC after excluding a variable
CREA	0.806	0.167
D_Dimer	0.901	0.072
TP	0.946	0.027
Ca	0.874	0.099
PDW	0.876	0.097
Ka	0.884	0.089
FIB	0.557	0.416

### Clinical Prediction Model

**FIGURE 2:**
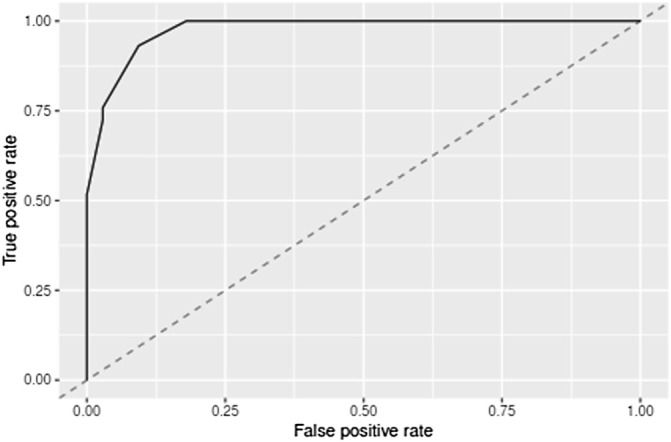
Cross-validated receiver operating characteristic curves for the model predicting tremors due to tacrolimus administered during the treatment of NS. AUC indicates the area under the receiver operating curves.

After tuning the single hidden layer NN model, the optimal parameter was eight. This implied that when the number of neurons in the hidden layer was 8, the prediction model exhibited optimal performance. The model was then updated and retrained based on the tuning results. The ROC of the prediction model is shown in Figure 2, and the corresponding AUC value was 0.9726, which, being close to 1, implied that the classifier performed well. The AUC value was often used as an additional performance index. The accuracy, sensitivity, and specificity of the training set were 0.9345, 0.9712, and 0.7586, respectively. These results sufficiently indicated that a good distinction between the NS patients under tacrolimus therapy with and without tremors was obtained from this prediction model. To verify the performance of the developed model, we tested it based on a dataset containing seven patients with tremors and 64 patients without tremors as external validation. The comparison between the basic information of the training and validation sets is shown in Table 4. The external validation achieved an accuracy of 0.8214, a sensitivity of 0.8378, and a specificity of 0.7000. The accuracy, sensitivity, and specificity of the training and validation sets are shown in Table 5. These high prediction metrics indicate that the tuned prediction model could predict tremors effectively.

**TABLE 4:** Basic information on the demographics and feature variables in the training and validation sets.

Variable	Train_mean	Train_conf	Test_mean	Test_conf	*p* value
Sex	69	181	29	71	0.799
Age (years)	44.249	2.031	45.662	3.154	0.462
PDW	12.726	0.299	12.431	0.484	0.302
TP	50.862	1.388	50.041	2.479	0.548
CREA	96.595	17.247	91.982	13.514	0.752
Ka	4.688	1.110	4.038	0.102	0.471
Ca	2.741	1.125	2.327	0.546	0.656
FIB	4.154	0.165	4.119	0.275	0.824
D_Dimer	469.221	120.527	378.014	101.869	0.375

**TABLE 5:** Prediction metrics of the training and validation sets.

	Training set	Validation set
Accuracy	0.9345	0.8214
Sensitivity	0.9712	0.8378
Specificity	0.7586	0.7000

## Discussion

NS is the most common clinical phenotype of immune-mediated glomerular diseases and is also the main cause of end-stage nephropathy (Gipson et al., 2016). Immunomodulatory agents, including glucocorticoids, are well-recognized therapeutic choices. In addition, calcineurin inhibitors (CNIs) suppress T cells and T-cell-dependent B-cell activation by blocking the phosphatase activity of calcineurin. CNIs such as tacrolimus are widely used for further immunosuppression in immune-mediated glomerular diseases, particularly in steroid-dependent and steroid-refractory NS (Floege et al., 2019). Although NS patients are treated with a smaller dose of tacrolimus than transplant patients, tremors are a major ADR of the nervous system that is often experienced by patients. Many patients with NS have to work and maintain a normal life, and the tremors that affected both the upper and lower limbs impacted the patients significantly.

In our study, the subjects clinically manifested NS. Even when the therapeutic dose of tacrolimus was maintained at less than 4 mg and the blood concentration was under 8 ng/ml, the occurrence of tremors was as high as 15.5%. The average age of patients was 42.5 years. Younger patients or those having greater demands at work may find it more difficult to deal with tremors. Unfortunately, there are few definitively effective strategies for prophylaxis or treatment of tremors caused by tacrolimus therapy. Therefore, it is necessary to establish a model that includes various factors related to patients that can prevent tremors, especially for those who might be at a higher risk of developing ADRs. One study also investigated the features (such as cholesterol plasmatic concentration) associated with the tremors caused by tacrolimus therapy; however, the results were also influenced by a single factor (cholesterol plasmatic concentrate), and further research needs to be conducted to evaluate the role of cholesterol plasmatic concentration in predicting the occurrence of tremors in patients under tacrolimus therapy (Erro et al., 2018). In this study, it was evident that seven variables (CREA, D_Dimer, TP, Ca, PDW, Ka, and FIB) might have been related to the occurrence of predicted tremors, and we focused on the importance of all the variables as a whole on the occurrence of tremors, rather than individual variables. This is more consistent with the complex clinical reality. What is the theoretical relationship between these variables and the occurrence of tremors? CREA may affect the excretion of drugs including tacrolimus. Less excretion of drugs can lead to an accumulation of drugs in the body and increases the incidence of adverse reactions. Ca and Ka are ions that play an important role in the functioning and maintenance of muscle nerve conduction in the body. The change in the levels of Ca and Ka may also cause some kind of tremor. Therefore, it was not surprising that CREA, Ca, and Ka were associated with the occurrence of tremors. The remaining indicators seemed to correlate with the state of coagulation in the body as a whole. It is worth noting that the clinical manifestations of NS patients are also hypoproteinemia and hypercoagulability. Therefore, we suspected that the occurrence of low-dose tacrolimus tremors is frequent, and the pathophysiological state of the NS (hypoproteinemia and high coagulation state) might have played a role. The effects of albumin and coagulation on drug entry into the body are mainly concentrated in drug distribution, especially for a high protein-binding drug (tacrolimus). Therefore, whether the special physiological state of tacrolimus affected its pharmacokinetic behavior remains to be studied. Meanwhile, further explanation of the influencing factors of tremors will depend on more experimental studies.

We have encountered many limitations in our study. A few basic characteristic variables might not have been included due to the lack of data such as blood lipids. Insufficient selection of categorical variables, drug interactions, and the use of a single-center study are a few other drawbacks of this study. The sample size of this study was small with a large number of variables, and this imbalance may have led to overfitting of the model, resulting in high accuracy. At the same time, the validation arm contains only a few patients with NS who developed tremors, and more patients are needed to be continued to better confirm the sensitivity and specificity of the algorithm. However, machine learning is a process that can be perfected by continuous training as new data are introduced. Anyway, this study offered one possible method for predicting tremors caused by tacrolimus in NS patients. The clinical application of this prediction model can identify the high-risk group of tremors early with a low dose of tacrolimus in patients with nephrotic syndrome and improve the treatment compliance of patients.

Many machine learning techniques have been widely used in medicine (Leung et al., 2013; Meng et al., 2019). RFE is popular machine learning technique because of its ease of configuration and use. RFE can be combined with different classifiers, such as NNs, support vector machines, and random forest (RF). Additionally, RFE is effective in selecting the features in a training set that are most relevant for variable prediction (Peng et al., 2019; Wottschel et al., 2019). In this study, a method combining RFE with RF was also developed to build a prediction model using the same data. RF was implemented by the RF function in R language (ver 3.6.3). It is an extension of the bagging method, which is a typical ensemble learning method. There are two main parameters in Rf: mtry (the number of variables randomly sampled as candidates at each split) and ntree (the number of trees to grow) (Rahman et al., 2017; Lai et al., 2020). In this model, every parameter of the optimal model can be tuned after the 10-fold cross-validation as follows: mtry was 9, ntree was 16, and nodesize (minimum size of terminal nodes) was 38. After calculation, the AUC value of the training set was 0.843, and the ACC value was 0.827. The external validation achieved an AUC value of 0.744 and an accuracy of 0.893. Evidently, the classification effect of the RF method was inferior to that of the NN. Notably, the number of tremors predicted by the RF method in the confusion matrix was 0. This implied that the RF method did not correctly predict tremors for the data used. The NN method was more suitable for our data based on the characteristics of the sample size and the variables collected.

## Conclusion

A risk prediction model with excellent predictive ability for tremors occurring during the treatment of NS patients using tacrolimus has been successfully established. This model can also be applied to patients who are administered with small doses of tacrolimus. It is the first model to include seven features: CREA, D_Dimer, TP, Ca, PDW, FIB, and Ka. However, this study has encountered many limitations, including the exclusion of a few basic characteristic variables due to the lack of data on features such as the level of blood lipids, insufficient selection of categorical variables, drug interactions, and the use of a single-center study. The imbalance between the small sample size and a large number of variables may have led to overfitting of the model, resulting in high accuracy.

## Data Availability

The raw data supporting the conclusions of this article will be made available by the authors, without undue reservation.
